# Glycemia-Induced miRNA Changes: A Review

**DOI:** 10.3390/ijms24087488

**Published:** 2023-04-19

**Authors:** Sara Al-Mahayni, Mohamed Ali, Muhammad Khan, Fatema Jamsheer, Abu Saleh Md Moin, Alexandra E. Butler

**Affiliations:** 1 School of Medicine, Royal College of Surgeons in Ireland Bahrain, Busaiteen 15503, Bahrain; 20204616@rcsi-mub.com (S.A.-M.); 18229425@rcsi-mub.com (M.A.); 19208132@rcsi-mub.com (M.K.); 16178092@rcsi-mub.com (F.J.); 2 Research Department, Royal College of Surgeons in Ireland Bahrain, Busaiteen 15503, Bahrain; amoin@rcsi.com

**Keywords:** miRNA, diabetes, hypoglycemia, hyperglycemia, complications

## Abstract

Diabetes is a rapidly increasing global health concern that significantly strains the health system due to its downstream complications. Dysregulation in glycemia represents one of the fundamental obstacles to achieving glycemic control in diabetic patients. Frequent hyperglycemia and/or hypoglycemia events contribute to pathologies that disrupt cellular and metabolic processes, which may contribute to the development of macrovascular and microvascular complications, worsening the disease burden and mortality. miRNAs are small single-stranded non-coding RNAs that regulate cellular protein expression and have been linked to various diseases, including diabetes mellitus. miRNAs have proven useful in the diagnosis, treatment, and prognosis of diabetes and its complications. There is a vast body of literature examining the role of miRNA biomarkers in diabetes, aiming for earlier diagnoses and improved treatment for diabetic patients. This article reviews the most recent literature discussing the role of specific miRNAs in glycemic control, platelet activity, and macrovascular and microvascular complications. Our review examines the different miRNAs involved in the pathological processes leading to the development of type 2 diabetes mellitus, such as endothelial dysfunction, pancreatic beta-cell dysfunction, and insulin resistance. Furthermore, we discuss the potential applications of miRNAs as next-generation biomarkers in diabetes with the aim of preventing, treating, and reversing diabetes.

## 1. Introduction

Diabetes mellitus is a chronic heterogenic metabolic disease that is characterized by an increased concentration of plasma glucose that results from a loss in pancreatic beta-cell mass and a defect in insulin secretion or action [1,2,3]. According to the International Diabetes Federation (IDF), 537 million adults between the ages of 20 and 79 years were living with diabetes in 2021. This number is predicted to rise to 643 million by 2030 and 783 million by 2046 [4]. The differentiation between type 1 diabetes (T1DM) and type 2 diabetes (T2DM) was only made in 1936 [2]. T1DM is an autoimmune disorder that causes the destruction of pancreatic beta-cells and is characterized by the inability to produce adequate insulin to meet the body’s physiological needs [1,5]. Conversely, T2DM is characterized by a combination of beta-cell loss through apoptosis, insulin resistance (IR), and relative insulin deficiency resulting from different genetic, behavioral, and environmental factors [2,6,7]. Poor glycemic control in T1DM and T2DM can lead to macrovascular and microvascular complications that are difficult to treat and significantly impact the diabetic patient’s life [2]. Even though conventional treatments for diabetes have made many advances in the last decade, a better understanding of molecular biology can assist in developing innovative therapeutics for this disease [5,6,7,8].

miRNAs play a vital role in the regulation of gene expression. Recent studies have examined their involvement in diabetes pathogenesis and complications [5,6,7]. Even though their role in the clinical management of diabetic patients remains largely unknown, recent studies have looked at their use as potential biomarkers for the diagnosis, management, and prognosis of diabetes. In this review, we discuss the role of specific miRNAs in glycemic changes associated with diabetes and their use as future biomarkers for diabetes [6,7].

## 2. The Pathophysiology of Diabetes Mellitus

Hyperglycemia is characterized by abnormally high blood glucose (>125 mg/dL (>7 mmol/L) while fasting and >180 mg/dl (>10 mmol/L) postprandially) [2,9,10]. Pre-diabetes or impaired plasma glucose (IPG) are characterized by a fasting plasma glucose (FPG) of 100–125 mg/dL (5.6–7.0 mmol/L), while diabetes is characterized by a fasting blood glucose over 125 mg/dL (>7.0 mmol/L) [2,9,10]. Diabetes is a complex, progressive, and challenging disease to treat with significant associated morbidity and mortality [1]. By the time the diagnosis is made, many patients already have significant complications [4,5]. T2DM can result in multisystem complications that can either be macrovascular, such as cardiovascular diseases and stroke, or microvascular, such as retinopathy, nephropathy, and neuropathy [4,5]. These complications can result in devastating consequences such as blindness, lower limb amputation, kidney failure, and death [4,5].

The pathophysiology of T2DM involves many systems and organs, including the pancreas, skeletal muscle, liver, kidney, brain, and intestines [4]. Chronic hyperglycemia can trigger inflammation, increased oxidative stress, lipotoxicity, and glucotoxicity, resulting in many pathologies such as dyslipidemia, hypertension, cancer, cardiovascular diseases, and infections [1].

A better understanding of the gene–environment risk factors for T2DM may allow for more timely interventions and the development of innovative targeted therapies to prevent or delay the onset of the disease [6]. Networks that regulate RNA and RNA binding protein (RBP) have been linked to various systemic manifestations of diabetes. miRNAs have been studied in relation to a number of metabolic diseases, including diabetes mellitus, where altered expression has been linked to different aspects of the disease and its complications [1,5].

## 3. The Pathophysiology of Hypoglycemia

Hypoglycemia is an important limiting factor and a significant obstacle in the glycemic management of diabetes. It is defined as “any abnormally low plasma glucose concentration that exposes the subject to potential harm” and is diagnosed by a plasma glucose reading below the threshold value of <70 mg/dL (<3.9 mmol/L) according to the American Diabetes Association (ADA) and the European Medicines Agency (EMA) [11,12,13].

Diabetic patients can experience hypoglycemic events from medication use (iatrogenic hypoglycemia) either due to insulin, oral anti-diabetic agents (OADs), or incretin-based therapies [13]. Other rare causes can include inborn errors of metabolism, starvation, extreme exercise, endocrine diseases, autoimmune diseases, tumors, infections, or dietary toxins [13,14]. Symptoms of hypoglycemia can either be neuroglycopenic, such as confusion, slurred speech, and seizures, or neurogenic, like sweating and palpitations [13,14]. Moreover, hypoglycemia can lead to coma or death if left untreated [13,14]. Over time, the body can adjust to recurrent hypoglycemic episodes, making the symptoms less pronounced and putting the patient at risk of hypoglycemia unawareness; this can place the diabetic patient at even higher risk of severe life-threatening hypoglycemia [13,14]. Strict glycemic control (defined as a glycosylated hemoglobin (HbA1c) <6.5% [48 mmol/mol]), as is advised based on multiple studies such as the Diabetes Control and Complications trial (DCCT) and the UK Prospective Diabetes Study (UKPDS), increases the risk of hypoglycemia approximately three-fold [15,16]. Repeated episodes of hypoglycemia, especially severe ones, are associated with increased morbidity and mortality in diabetic patients [13].

Glucose is the primary energy source for the brain, nervous system, and erythrocytes due to the inability of these tissues to store glucose for fuel during starvation [15,16]. The disruption of the cerebral glucose supply causes the neuroglycopenic symptoms associated with hypoglycemia [11,14,17]. Hypoglycemia activates different brain regions, such as the hypothalamus and medial prefrontal cortex [17]. Hypoglycemia-associated autonomic failure (HAAF) is a maladaptive response that can be caused by recurrent episodes of insulin-induced hypoglycemia (RH) wherein there are impaired glucose sensing mechanisms in the brain and the glucose counterregulatory response (CRR) [14,17]. RH induces HAAF through physiological mechanisms such as counterregulatory hormones (cortisol, epinephrine, and opioids) or behavioral issues such as hypoglycemia unawareness [11,18]. Impairment in the CRR results in an inadequate epinephrine response to decreasing glucose levels and an impaired glucagon secretory response. By contrast, hypoglycemia unawareness decreases the autonomic response to hypoglycemia and decreases the sympathoadrenal response causing neurogenic symptoms [12]. Moreover, other mechanisms causing HAAF include increasing gamma-aminobutyric acid (GABA) tone, expanding the use of alternative brain fuels such as ketone bodies, and increasing glycogen storage in the brain [18].

Repeated episodes of severe hypoglycemia can also aggravate neurocognitive dysfunction in older adults and increases the risk of developing dementia, cerebellar ataxia, and functional brain failure [11,13].

Hypoglycemia impacts cardiac function, with severe episodes potentially causing cardiac death due to ischemia or depolarization/repolarization changes [11,13]. The release of epinephrine due to sympathetic and adrenal activation following an episode of hypoglycemia can increase the heart rate and peripheral systolic blood pressure while reducing central blood pressure and peripheral arterial resistance which, in turn, increases stroke volume, contractility, and cardiac output [11,13].

Hypoglycemia has been linked to visual changes and disorders in diabetic patients, such as blurred vision, diplopia, increased retinal degeneration, and the loss of contrast sensitivity and central retinal function [13].

Additionally, hypoglycemia can impact the diabetic patient’s life by provoking anxiety, hopelessness, depression, social isolation, impaired task performance, and sleep disturbances [13]. Furthermore, hypoglycemia can provoke dietary overcorrection with increased overall carbohydrate intake, leading to weight gain and poor glycemic control [13].

Recent research has shed light on the role of miRNAs in the control of glucose homeostasis through their control of gene expression for critical biological processes [11]. miRNAs have a role in the regulation of hypoglycemia, a complication that is responsible for major morbidity and mortality in the diabetic population [11]. Moreover, miRNAs have been linked to various pathologies associated with hyperglycemia that are explored in this review [5].

## 4. miRNA Biology

microRNAs (miRNAs and miRs) are small single-stranded non-protein coding RNAs that are ~22 nucleotides in length and are endogenously expressed [19]. They were discovered in 1993 by the Ambros and Ruvkun groups in Caenorhabditis elegans, a revolutionary discovery in the field of molecular biology [19]. The Human Genome Project reported that around 1000 genes encode for miRNAs, constituting ~3% of the entire human genome [19]. miRNAs are first transcribed from DNA sequences into primary (pri)-miRNAs containing one or more hairpin structures that then are processed into precursor (pre-)miRNAs and mature miRNAs [19,20]. Biogenesis begins post or co-transcriptionally with RNA polymerase II/III processing and can either be intragenic or intergenic through both canonical and non-canonical pathways [20,21]. miRNA biogenesis is tightly regulated by epigenetic and transcriptional factors defining the different miRNAs at the cellular level [21]. miRNA efficiency can be affected by transcription factors, such as the tumor suppressor protein p53, or proteins affecting mRNA stability, such as the KH-type splicing regulatory protein (KSRP) [7,11,20,21].

miRNAs play a vital role in directing cellular fate through cellular signaling, proliferation, differentiation, homeostasis, and apoptosis [7,21]. They cause transcriptional repression and enhanced mRNA target degradation through recognition of complementary, or partially complementary, sequences on the 3′ untranslated region (3′-UTR), which can affect protein synthesis [7,21]. miRNAs can interact with 5′ UTR, gene promoters, and the coding sequence, and they can be shuttled between various subcellular compartments, thereby affecting both translation and transcription [21,22,23]. miRNAs play a critical role in regulating physiological and pathological processes within the cell by controlling mRNA stability and translation [7,21]. Additionally, they can control multiple genes by impacting gene expression at the post-transcriptional level, affecting a wide array of metabolic processes [21]. Extracellular miRNAs can serve as potential biomarkers for diseases [21,23].

Alterations in miRNA expression have been linked to multiple pathological conditions such as type 2 diabetes, cancer, periodontal and autoimmune diseases [11,20]. Oncogenic miRNAs (oncomiRs) and tumor suppressor miRNAs can play a role in cancer progression, prognosis, and treatment [11,20]. Additionally, miRNA expression can promote viral replication in the host cell [11,20]. miRNAs can play a role in preventing autoimmune diseases like rheumatoid arthritis and systemic lupus erythematosus and serve as biomarkers for some autoimmune diseases. Autoantibodies can be targeted by proteins involved in miRNA biogenesis, such as Argonaute 2 (Ago2) [11,20,22]. Furthermore, miRNAs in the hypothalamus have been linked to dietary and energy intake, leading to IR when their expression is dysregulated [11,20,22].

## 5. Relationship between Glycemic Changes and miRNAs

### 5.1. Hypoglycemia-Induced miRNA Changes

Hypoglycemia can induce significant miRNA changes affecting the hypothalamic neurons [11]. MicroRNA-7a-5p can downregulate miR-655 through miRNA degradation or translational repression and by interacting with the 3′ UTR of the target genes [11]. Moreover, miRNA-7a-5p is predicted to bind specifically to the Gabra1 3′-UTR, negatively regulating *Gabra1* gene expression (the gene that encodes the Gamma-aminobutyric acid receptor subunit alpha-1 protein) at a post-transcriptional level [11].

miR-155 is required to help maintain normal glucose levels in physiological conditions as it targets the *B-cell integration cluster* gene (Bic) [24]. The *Bic* gene is found in both mice and humans and has been shown to positively relate to hypoglycemia and improved sensitivity to insulin and glucose uptake [24]. Furthermore, insulin receptor substrate 1 (IRS-1) phosphorylation and insulin-stimulated serine/threonine kinase (AKT) are increased by the overexpression of miR-155 in mouse liver, adipose tissue, and skeletal muscle [25].

miR-155 was found at the binding site of the four target genes involved in the upregulation of blood glucose and sensory impairment, namely *CCAAT/enhancer-binding protein beta (C/EBPβ)*, *Histone Deacetylase 4 (HDAC4)*, *the suppressor of cytokine signaling 1 (SOCS1)*, and *pyruvate dehydrogenase kinase 4 (PDK4)* [26]. miR-155 negatively regulated the expression of these four target genes leading to hypoglycemia, improved glucose tolerance, and increased sensitivity in peripheral tissues; in addition, the lack of miR-155 had the opposite effects [26].

A recent study looking at serum differential miRNA expression in T2DM patients after one hour of iatrogenic induction of hypoglycemia by intravenous (IV) insulin infusion showed that certain miRNAs were up-regulated and down-regulated by hypoglycemia [25,27]. Interestingly, however, the altered expression of miRNAs was restricted to control subjects and not the T2DM patients, suggesting that the protective effect of these miRNAs may be lost in T2DM [25,27]. Multiple miRNAs were upregulated (miR-let-7e-5p, miR-1303, miR-1267, miR-571, miR-661, miR-770-5p, miR-30a-5p, and miR-892b) whilst only one was downregulated (miR-652-3p) in the control subjects, and those miRNAs that exhibited the most significant change in differential expression were all linked to disease processes in the T2DM patients, strongly indicating a specific relationship between diminished miRNA responses and particular pathological processes in T2DM [27].

A follow-up study on the same population found that miRNAs were up-regulated and down-regulated up to 24 h after hypoglycemia, likely due to the action of certain counter-regulatory hormones. miR-191-5-p, miR-143-3p, and let-7g-5p were shown to be associated with the counterregulatory response and with energy and glucose homeostasis [2]. These miRNAs were downregulated in both the controls and the T2DM patients at 24 h [2]. For future diagnostic utility, the potential exists to design a panel of miRNAs as biomarkers to aid in the diagnosis of hypoglycemia.

### 5.2. Hyperglycemia-Induced miRNA Changes

T2DM is characterized by hyperglycemia due to a combination of IR and relative insulin deficiency that results from a combination of genetic, environmental, and lifestyle factors [2]. Many factors play a vital role in glucose homeostasis, including pancreatic beta-cell function, insulin signaling, incretin release, and glucagon secretion [2]. Several studies suggest that hyperglycemia alters a specific set of miRNAs, reflecting alterations within the intracellular machinery and reflective of the order in which each alteration occurs [2,10].

#### 5.2.1. Endothelial Dysfunction

Endothelial dysfunction is a very early pathogenetic step in hyperglycemia-induced macrovascular disease [28,29]. It has been reported that, under hyperglycemic conditions, endothelial cells experience a significant reduction in nitric oxide (NO) release, which occurs in conjunction with a significant increase in endothelial miR-24 [28,29,30]. Further investigation has revealed that miR-24 transcripts bind directly to the 3′UTR region of the *nitric oxide synthase 3 gene (NOS3)*, reducing nitric oxide synthase expression [28,29,31,32,33]. Interestingly, mutations within miR-24 that compromise its complementarity with NOS3 transcripts have been associated with increased NO and may exert protective actions [34].

#### 5.2.2. Pancreatic Beta-Cell Dysfunction

miRNAs play a vital function in glucose homeostasis by affecting insulin secretion through the regulation of pancreatic beta-cell proliferation, differentiation, apoptosis, and overall function. miR-22 overexpression has been linked to beta-cell apoptosis through activation of the *Bax* and *Trp53* apoptotic genes [35,36,37,38,39]. miR-34a can cause mouse insulinoma 6 (MIN6) cell apoptosis by binding to Bcl-2 on its 3′-UTR [40]. Multiple miRNAs, one example being miR-375, can affect beta-cell proliferation; miR-375 is expressed in pancreatic beta-cells and targets multiple growth-inhibiting genes [39,40]. A decrease in this miRNA can contribute to impaired pancreatic beta-cell proliferation [41]. Moreover, miR-17 can promote beta-cell proliferation, while miR-24 and miR29a can inhibit it [2].

**Figure 1:**
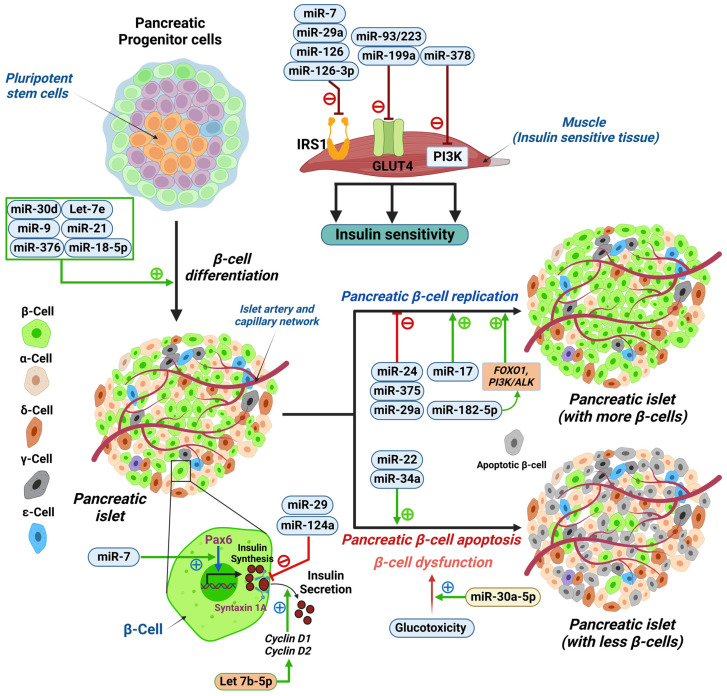
Schematic illustration showing micro RNAs (miRNAs) involved in pancreatic β-cell differentiation, function, and survival. miRNAs that regulate insulin action in insulin sensitive tissues (muscle, for example) are miR-7, miR-29a, miR-126, and miR-126-3p that negatively regulate IRS1; miR-93/223 and miR-199a that negatively regulate glucose transporter type 4 (GLUT4); and miR-378 that negatively regulates phosphoinositide 3-kinase (PI3K). miR-30d, let-7e, miR-9, miR-21, miR-376, and miR-182-5p positively regulate pancreatic β-cell differentiation from pancreatic progenitor cells. While miR-24, miR-375, and miR29a negatively regulate pancreatic β-cell replication, miR-17, and miR-182-5p (by activating the FOXO1, PI3K/ALK pathway) is a positive regulator. miR-22 and miR-34a induce pancreatic β-cell apoptosis. miR-30a-5p modulates the glucotoxicity induced β-cell dysfunction. miR-7 positively regulates Paired box protein (Pax-6) to enhance insulin synthesis in β-cells. Let-7b-5p positively regulates insulin secretion by modulating cyclin D1 and cyclin D2. miR-29 and miR-124a negatively regulate syntaxin 1A, which reduces insulin secretion from pancreatic β-cells. Pancreatic islets contain insulin producing (β-cells), glucagon producing (α-cells), somatostatin producing (δ-cells), pancreatic polypeptide producing (Ɣ-cells), and ghrelin producing (Ɛ-cells) endocrine cells. FOXO1, Forkhead box protein O1; PI3K, phosphoinositide 3-kinase; ALK, anaplastic lymphoma kinase. Illustration created using Biorender.com (with publication license).

Beta-cell differentiation is affected by multiple miRNAs, such as miR-375, that can downregulate HNF1β when overexpressed, while miR-7 can decrease Paired box protein 6 (PAX-6), making them both essential in beta-cell differentiation [42]. Other miRNAs involved in differentiation include miR30d, let-7e, miR-21, miR-9, and miR-376 [42]. Furthermore, insulin secretion is affected by miRNAs, such as miR-29, that target *Syntaxin-1A (stx-1a)* leading to negative regulation of insulin exocytosis [43]. Other miRNAs that can negatively affect insulin secretion from pancreatic beta-cells include miR-124a, which targets Guanosine triphosphatase Ras-associated binding protein 27a (GTPases Rab27a) and Forkhead box protein A2 (FOXA2) promoting T2DM through beta-cell dysfunction (Figure 1) [44,45]. Studies have linked miR-182-5p to glucose regulation via its targeting Forkhead box protein O1 (FOXO1), which has an essential role in beta-cell replication and differentiation in the pancreas [46]. During hyperglycemia, miR-182-5p plays an essential role in suppressing gluconeogenesis by affecting insulin-like growth factor-1 (IGF1) and its receptor and subsequently stimulating the phosphoinositide-3-kinase-protein kinase/akt (PI3K/Akt) signaling pathway and FOXO1 [46]. This study also showed that miR-128-5p levels decrease with an increased duration of diabetes [46]. Furthermore, miR-30a-5p was found to be linked to dysfunction of ß-cells in the pancreas by suppressing the expression of the *Beta 2/neurogenic differentiation D1 (BETA2/NeuroD)* gene leading to glucotoxicity. This miRNA was suggested as a potential biomarker for the prediction of the development of T2DM as higher levels were observed in newly diagnosed T2DM patients in the first year after diagnosis compared to non-diabetic patients [46].

#### 5.2.3. IR

Insulin binds to the insulin receptor on the surface of the cell, causing a signaling cascade that includes AKT, GLUT-4, and PI3K [2]. Studies show that miR-195 and miR-15b can downregulate insulin receptors (INSRs), disrupting insulin signaling in hepatocytes [8]. miRNAs can also modulate the expression of IRS-1, which translates the signal from the INSR into the downstream enzymatic cascade [47]. miR-7 can downregulate IRS-1 through 3′UTR binding, while miR-96 can be upregulated in IR [47]. Furthermore, miRNAs seem to play a role in diet-induced IR. miR-29a expression, for example, has been linked to IR through repression of IRS-1 in diets rich in saturated fats [48]. Similarly, miR-126 correlates negatively with IRS-1 in maternal diet-induced obesity in offspring [49]. mir-126-3p is a marker of glycemic dysregulation as it plays a vital role in insulin signaling in hyperglycemic states [46]. Its overexpression was linked to a decrease in IRS-1 in smooth muscle cells [46]. Another study focused upon let7b-5p and its link to insulin regulation, revealing that the overexpression of let7b-5p was associated with increased insulin content and increased insulin secretion in response to glucose through the decreased expression of Cyclin D1 and Cyclin D2 and the inhibition of ß -cell proliferation [50,51]. Another study found that miR-421 was decreased in diabetic patients compared to controls, especially in patients with a normal or overweight body mass index (BMI) [52]; this miRNA was linked to obesity as well as to angiotensin-converting enzyme 2 (ACE2) regulation, which has been linked to viral infections such as SARS-CoV-2 infection [52].

Moreover, some miRNAs, such as miR-021, miR-27b, miR-103, and miR-155, have been linked to IR and polycystic ovary syndrome (PCOS) indirectly through inflammatory pathways such as tumor necrosis factor (TNF) and interleukin-6 (IL6) [9]. In contrast, miRNA-1260a has been linked to a high free androgen index (FAI) [9]. Other miRNAs are involved in the enzymatic downstream cascade, such as miR-378, which inhibits insulin signaling by targeting PI3K, and miR-199a and miR-93/223 directly impact GLUT4, thereby decreasing insulin sensitivity [53,54] (Figure 1).

## 6. The Implications of miRNAs in Hypoglycemia

miRNAs are potentially promising for use as diagnostic agents and for therapeutic intervention because of their molecular properties, particularly their secretion into extracellular fluids and their capacity to regulate gene expression [21,55]. Although their role in hyperglycemia and T2DM has received considerable attention, their role in hypoglycemia remains relatively unexplored [55].

In recent years, there have been some reports, albeit few, of hypoglycemia-associated miRNA changes that hold the potential to translate directly into clinical practice, most prominently within the context of HAAF [55,56]. This is particularly opportune, given that current methods of detecting, managing, and preventing the occurrence of HAAF are notably inadequate, causing a significant proportion of the diabetic population to experience severe life-threatening hypoglycemic episodes [21,55].

The hypothalamic ventromedial nucleus (HMVN), believed to be the primary initiator of the sympathoadrenal response defective in HAAF, is reported to abundantly express miR-7a-5p, which is thought to influence post-transcriptional regulation of MAPK-interacting serine-thionine kinase 2 [18,57]. The investigation of HAAF-related miRNA changes in the HMVN of rodents through recurrent artificial HVMN glucopenia [11,12] demonstrated significant impairment in the sympathoadrenal response, reduced miR-7a-5b levels, and increased synthesis of glutamic acid decarboxylase 65 and GabaA1 receptor subunits in animal models [18,57]. Lentivirus-mediated restoration of miR-7a-5p levels yielded almost complete restoration of the sympathoadrenal response with peak serum epinephrine levels correlating with HMVN miR-7a-5b levels [18,57]. Further investigation revealed that miR-7a-5b directly binds to the 3′-UTR region of *Gaba-1* gene transcripts, leading to reduced HVMN sensitivity to surrounding inhibitory signals [11,57]. This provides significant insight into the pathophysiological mechanisms that govern HAAF, specifically that the attenuation of the ventromedial medulla, the primary structure involved in initiating the CRR, occurs due to metabolic changes that lead to increased responsiveness to inhibitory signals generated by recurrent hypoglycemia [11,12,18,57].

A similar study investigated the influence of hypoglycemic environments on the expression of Fos Proto-Oncogene, AP-1 Transcription Factor Subunit (FOS), and Fat Mass and Obesity-Associated Gene (FTO) proteins, as well as differential miRNA change, in rodent embryonic hypothalamic neuron cultures (EHN) [22]. FOS protein expression within hypothalamic tissue has been reported to be significantly suppressed under hyperglycemic conditions, suggesting its involvement in glucose homeostasis [22]. Conversely, hypoglycemia significantly increased FOS expression and reduced FTO expression. Notably, these changes were associated with a reduction in miR-7a and miR-9 amongst other miRs [22]. Administration of anti-miR-9 markedly reversed the expression of FOS under hypoglycemia; however, it remains unclear whether FOS expression enhances EHN viability in response to hypoglycemia [22].

Collectively, changes in hypothalamic miR-7a-5p and miR-9, among others, appear to be indicative of the presence of HAAF in diabetic patients [11,22]. These alterations could be critical in clinically characterizing and preventing its occurrence, specifically via generating a serum/body fluid-based miRNA profile consisting of significantly reduced miRNA-7a-5p and miR-9 within a range predictive of HAAF presence and necessitating clinical intervention [22]. Additionally, the therapeutic reversal of these changes may be achieved through pharmacological manipulation of miR-7a-5p and miR-9 levels [11,22].

No clinical studies, however, have investigated the reliability of serum-based miRNA detection in identifying HAAF, specifically whether miR-7a-5p and miR-9 are secreted in a pattern that reflects the status of HMVN neurons and whether these miRNAs are exclusively secreted from HMVN, as it is a possibility that other remote cells may contribute to total body fluid levels and hence weaken its validity [26]. Given this possibility, further investigation is required to identify miRNAs that undergo a similar change in expression and, therefore, can be used in conjunction with the already identified miRNAs, as a panel of miRNAs is always likely to be diagnostically more robust [26].

Therapeutically targeting hypothalamic miR-7a-5p and miR-9 is likely more challenging, given the restrictive nature of miR-based therapy [26]. This is mainly attributable to their limited pharmacokinetics, which includes the need to avoid phagocytosis, lysis via endonucleases, binding to serum proteins, inactivation by the liver, and renal clearance, all of which contribute to a short half-life [26]. Additionally, specific therapeutic options such as viral vectors raise safety concerns, among them the risk of nonspecific reactions, oncogenesis, and immunogenetics [26].

## 7. Future Directions

### 7.1. miRNAs as Potential Biomarkers for T2DM

The relationship between miRNAs and T2DM pathophysiology is complex, and multiple studies are investigating their use in clinical practice as biomarkers for diagnosis, prognosis, monitoring, and treatment of T2DM [2,58,59,60]. miRNAs are considered to be suitable biomarkers because they can be present intracellularly or extracellularly, allowing their detection in various physiological fluids depending on varying pathophysiological conditions [60,61,62,63]. Furthermore, they can be detected using multiple methods and are considered to be remarkably stable [60].

One of the earliest studies looking at the link between miRNAs and glucose metabolism in T2DM found a significant difference in the expression of miRNAs between T2DM patients and T2DM susceptible patients [62]. This study found that specific miRNAs (miR-9, miR-29a, miR30d, miR-34a, miR124a, miR146a, and miR-375 are involved in insulin metabolism and pathogenesis in T2DM [59]; these miRNAs were potentially reliable disease-specific markers as they only changed in T2DM patients and did not change drastically in pre-T2DM [59].

miRNAs appear to be linked to different pathophysiological aspects of T2DM, such as insulin receptor function, signaling, beta-cell function, IR, and glycemic control [64,65]. miR-126 has been identified as a potential early marker for the development of T2DM, as its expression was linked to the likelihood of developing T2DM over a two-year follow-up period [66,67,68,69]. Moreover, miR-320b, miR-1249, and miR-572 have been identified as potential biomarkers for early T2DM, as they were differentially expressed in prediabetic and recently diagnosed T2DM [2,68].

### 7.2. Biomarkers in T2DM Macrovascular Complications

The macrovascular complications of T2DM can manifest as vascular disorders in cardiovascular diseases, where atherosclerosis can be the main culprit [69,70,71]. Diabetes worsens atherosclerotic lesions in vessels, accelerating plaque formation and potentially leading to complications such as myocardial infarction (MI) [72,73,74,75].

Moreover, miR-126 has been studied as a potential biomarker for CAD [2]. miR-130 is a potential biomarker in CAD, distinguishing T2DM with CAD from non-diabetic CAD patients through regulating the expression of PPAR-γ [2,76]. In addition, miR-126 and miR-210 distinguish T2DM and T2DM with CAD from healthy patients; miR-210 showed decreased levels in T2DM patients with CAD, while miR-126 showed increased levels in diabetics and an even more significant increase in T2DM patients with CAD. Moreover, miR-1 and miR-133 have been linked to the early detection of cardiomyopathy in T2DM [77].

miR-483-3-p has a vital role in endothelial integrity in T2DM and has been proposed as a potential prognostic and therapeutic target in CAD in T2DM patients [77,78]. The miRNAs that are involved in macrovascular complications are summarized in Table 1.

### 7.3. Biomarkers in T2DM Microvascular Complications

miRNAs, such as miR-21, miR-29a/b/c, and miR-192, can be potential biomarkers for diabetic microvascular complications, such as diabetic nephropathy (DN) [67]. DN is a serious diabetic complication and the leading cause of end-stage kidney disease [73]. miR21-5p, miR-30b-5p, and miR-196a have been shown to have potential as biomarkers of renal function [74]. miR-196-a and miR-121 have been reported to be potential prognostic biomarkers of renal fibrosis in patients with DN [72,79]. miR-429 was associated with proteinuria, while miR-126 and miR-770 have been downregulated in the urine of diabetic patients, suggesting that they may all be involved in renal dysfunction in diabetic patients [79]. miR-200 and miR-29 downregulation have been linked to kidney dysfunction, indicating a possible protective effect of these miRNAs [79]. mir-126-3p has been linked to renal dysfunction in diabetic patients in multiple studies [46,80,81]. The overexpression of this miRNA has been linked to DN in long-standing diabetes [81]. Furthermore, some biomarkers have the potential for monitoring the progression of DN, such as miR-320c [72]. Some mechanisms through which these miRNAs impact DN are phosphatase and tensin homolog (PTEN) suppression, zinc finger E-box binding homebox2 (ZEB2) suppression, and Sprouty homolog 1 (SPRY1) inhibition, such as in the case for miR-21, miR-192, and miR-29c, collectively [71].

Moreover, miR-128a, miR-155, and miR-499 have been linked to neuropathic complications in T2DM [73]. miR-199a-3p has been linked to diabetic neuropathy progression, while miR-146 was found in inflammatory cells in patients with peripheral neuropathy [79]. Recent studies have shown that some miRNAs can have a vital role in mediating inflammation, Schwann cell myelinogenesis and proliferation, and axonal regeneration, making them good potential biomarkers for diabetic neuropathy in the future.

Diabetic retinopathy (DR) is a complex and challenging T2DM complication that accounts for around 80% of legal blindness in adults aged 20–74 [74,75]. Current treatments, such as anti-vascular endothelial growth factor (VEGF), have many limitations, prompting the investigation into miRNAs as potential diagnostic biomarkers and therapeutic targets [82]. miR-21, miR-320-a, and miR-320-b have been shown to have high sensitivity and specificity for retinal diseases as they have been found in postmortem vitreous humor [79]. miR-1281 has been linked to the early detection of DR in T2DM patients and has a possible pathogenic role in DR development in T2DM patients with poor glycemic control [83]. miRNAs such as miR-320-a and miR-27b interact with thrombospondin-1 and VEGF, making them important clinically [79]. In the same study, let-7a-5p was associated with increased retinal proliferation [79]. miR-27b and miR-320a have angiogenic effects by inhibiting VEGFc, which has been linked to the pathogenesis of DR [79]. Moreover, miRNA-195 has been linked to the regulation of sirtuin 1 (SIRT1), which becomes oxidized in DR, giving this miRNA importance in the management of DR [82]. miRNAs also have a role in diagnosing DR in T2DM patients where some have increased expression, such as miR-25-3p and miR-320b, while others have decreased expression in DR patients, such as miR-495-2p, versus T2DM patients without DR [84]. Furthermore, miR-21 was found to be indicative of the severity of DR in T2DM patients as its expression was correlated with disease severity, disease progression, HbA1c, FPG, and Homeostatic Model Assessment for Insulin Resistance (HOMA-IR) [41].

The miRNAs that are involved in microvascular complications are summarized in Table 2.

## 8. Conclusions

Our review provides a comprehensive overview of the role of miRNAs in glycemic deviations, discussing their involvement in hyperglycemia and hypoglycemia and the implications related to their differing levels of expression. Moreover, we discuss the link between different miRNAs and diabetes pathologies and complications, including the most frequent macrovascular and microvascular complications. We examined the current miRNAs that could be potentially useful as next-generation markers in the clinical management of diabetes to decrease the disease burden and provide innovative treatments. This review summarizes our improved understanding of the miRNA changes that lead to IR, prolonged hyperglycemia, endothelial dysfunction, and excessive platelet activation. By contrast, only a small number of studies have examined the role of miRNAs in hypoglycemia. However, the few presented here offer promising results that have the potential to be immediately implemented in clinical settings, emphasizing the need for additional research to further clarify their function and role in diabetes management.

## Figures and Tables

**Table 1:** The miRNAs that are involved in macrovascular complications.

Biomarker	Sample	Diagnostic Value	Reference
miR-126	Peripheral whole blood	Differentiates between T2DM with CAD from T2DM	[77]
miR-130	Serum	Differentiates between T2DM with CAD from CAD patients	[76]
miR-210	Plasma	Differentiates between T2DM with CAD from T2DM	[77]
miR-483-3p	Peripheral blood mononuclear cell (PBMC)	Differentiates between T2DM with CAD from CAD	[77,78]

**Table 2:** The miRNAs that are involved in microvascular complications.

Biomarker	Sample	Diagnostic Value	Reference
miR-21	Plasma	Specify severity of diabetic retinopathy in T2DM patients	[85]
miR-21-5p	Urinary exosome	Indicator of renal function	[74]
miR-30b-5p	Urinary exosome	Indicator of renal function	[74]
miR-196a, miR-121	Urine	Prognostic marker for renal fibrosis patients with DN	[72]
miR-320b, miR25-3p and miR-495	Plasma exosomes	Useful for diagnosing patients with diabetic retinopathy in T2DM	[84]
miR-128a, miR-155 and miR-499	PBMCs	Monitoring progression	[73]
miR-1281	Serum	Early diagnosis of diabetic retinopathy in T2DM patients	[83]
miR-320c	Peripheral blood	Monitoring progression of DN	[72]
miR-192	Peripheral blood	Early detection of T2DM DN	[70]
miR-29a/b/c	Peripheral blood	Potential biomarker, especially miR-29a	[67]
miR-429	Plasma	Potential marker for proteinuria and kidney function	[79]
miR-126, miR 29	Peripheral blood	Indicator of renal dysfunction	[79]
miR-199a-3p	Plasma	Indicator of neuropathy progression	[79]
miR-146	Plasma	Linked to peripheral neuropathy	[79]
miR-21, miR-320-a, mir-320-b	Post-mortem vitreous humor	Linked to DR development and pathogenesis	[79]
let-7a-5p	Serum	Linked to retinal proliferation	[79]

## Data Availability

Not applicable.
